# Athlete Hydration: Beyond Performance Toward Long-Term Health

**DOI:** 10.1007/s40279-026-02440-5

**Published:** 2026-04-22

**Authors:** Rúben Francisco, Lawrence E. Armstrong

**Affiliations:** 1 Sport Physical Activity and Health Research Innovation and Technology Center (SPRINT), Santarém Polytechnic University, Av. Dr. Mário Soares n.º 110, 2040-413 Rio Maior, Portugal; 2 Korey Stringer Institute, University of Connecticut, Storrs, CT USA

## Abstract

Research on fluid-electrolyte balance in athletes has largely emphasized acute hydration strategies to preserve performance, particularly under conditions of prolonged exercise and heat stress. While this focus has yielded valuable practical guidance, it has overshadowed the potential long-term health implications of habitual low water intake (LWI). Emerging evidence from non-athletic populations indicates that chronic LWI, often characterized by persistently elevated arginine vasopressin (AVP) and concentrated urine, may increase renal, endocrine, and metabolic strain, showing associations with insulin resistance, low-grade inflammation, and chronic disease risk. Notably, evidence suggests that up to 40% of the non-athletic population fail to meet the fluid intake recommendations. Furthermore, a considerable proportion of athletes are habitually classified as low drinkers (i.e., ~ 58% consuming < 35 mL·kg^−1^·day^−1^), despite being exposed to recurrent and substantial fluid and electrolyte losses during training and competition. Although total body water may remain within normal ranges in these individuals, markers of renal concentrating stress suggest sustained activation of fluid-regulatory systems. Whether this physiological state poses long-term health risks for athletes remains unknown. This short communication argues that habitual LWI represents an underappreciated and potentially modifiable health risk in athletic populations. We highlight critical gaps in longitudinal and mechanistic research, calling for a paradigm shift in sports nutrition that recognizes total daily water intake as both a performance variable and a determinant of long-term health.

## Key Points

**Table:** 

Research on athlete hydration has focused mainly on short-term performance, but many athletes regularly consume low amounts of water throughout the day. Although their total body water often appears normal, this may mask ongoing strain on the kidneys and hormonal systems that regulate fluid balance.
Evidence from non-athletic populations suggests that habitually low water intake may be linked to a higher risk of kidney problems, metabolic disturbances, and chronic disease. This article argues that similar long-term (e.g post-career) health risks could exist in athletes, even if performance is not immediately affected.
The authors call for a shift in sports nutrition research to examine total daily water intake as both a performance factor and a long-term health determinant. They highlight the need for long-term studies to determine whether increasing daily water intake can reduce potential renal and metabolic risks in athletic populations.

**Fig. 1:**
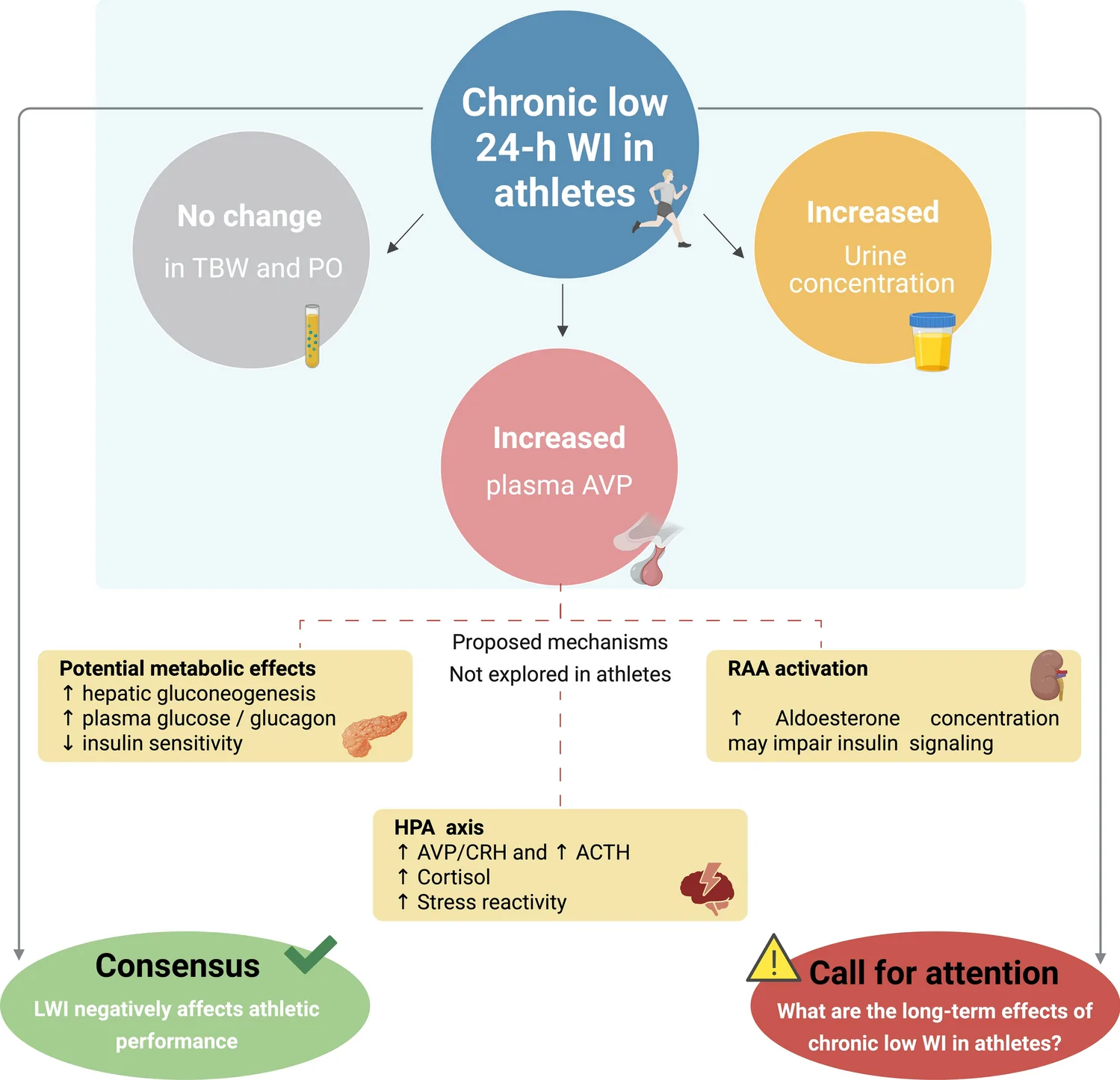
Conceptual framework illustrating how habitual 24-h low water intake in athletes may be associated with chronic endocrine and renal adaptations that preserve body water but potentially increase long-term renal and metabolic health risks. Although total body water and extracellular osmolality may remain within normal ranges, sustained renal concentrating stress and elevated plasma vasopressin may represent underappreciated mechanisms linking low water intake to long-term health outcomes. *LWI* low water intake, *WI* water intake, *TBW* total body water, *PO* plasma osmolality, *AVP* arginine vasopressin, *CRH* corticotropin-releasing hormone, *ACTH* adrenocorticotropic hormone, *HPA axis* hypothalamic–pituitary–adrenal axis, *RAA* renin–angiotensin–aldosterone system. Figure created in BioRender. Francisco, R. (2026) https://BioRender.com/jl9ay3p

Fluid-electrolyte balance in athletes has been a central topic in sports nutrition and exercise physiology over recent decades [1, 2]. The majority of research has focused on the acute effects of fluid deficits on thermoregulation, cardiovascular strain, and exercise performance, particularly during prolonged exercise and environmental heat stress [2]. This performance-oriented approach has generated important insights and practical recommendations. However, it has also fostered a relatively narrow conceptual framework in which fluid intake is viewed primarily as a means to preserve sport performance. While performance is undeniably important in athletic competition, it represents only one dimension of an athlete’s long-term trajectory. This short communication argues that habitual 24-h low water intake (LWI; i.e., daily water intake below recommended levels) may represent an underappreciated long-term health risk in athletes. It highlights the need for longitudinal and mechanistic research to address this gap, especially for those who experience moderate-to-severe fluid and electrolyte losses during training and competition. This conceptual framework, illustrating the potential physiological adaptations to chronic LWI and their possible implications for long-term health, is summarized in Fig. 1.

The long-term physiological consequences of habitual LWI have received limited attention, despite the central role of fluid intake in renal, metabolic, and hormonal regulation [3]. Evidence suggests that up to 40% of the non-athletic population appears to live in a state of chronic underhydration, characterized by LWI and persistently elevated plasma arginine vasopressin (AVP) concentrations [4]. In non-athletic populations (adults, 25–40 years old), “low drinkers” have been operationally defined using absolute thresholds, such as fluid intakes ≤ 1.2 L·day^−1^ (i.e., sum of drinking water and any other source of fluid) [5] or < 2.0 L·day^−1^ [6] in other frameworks, although these definitions do not account for body size or activity-related water losses. In contrast, Francisco et al. [7] defined LWI in athletes using a relative threshold of < 35 mL·kg^−1^·day^−1^, thereby accounting for inter-individual differences in body mass and providing a more context-specific criterion for athletic populations. Although this physiological state maintains total body water and plasma osmolality within narrow ranges, it may do so at the cost of increased endocrine and renal stress. In non-athletic populations, chronic LWI has been associated with kidney stone formation, insulin resistance, low-grade inflammation, mood disturbances, and increased risk of chronic disease [3, 8]. While causality has yet to be firmly established in published literature, the consistency of these associations warrants careful consideration. AVP plays a central role in this discussion. Beyond its antidiuretic function, elevated circulating AVP has been shown to promote hyperglycemia, stimulate hepatic gluconeogenesis, and increase plasma glucagon concentration [9]. Individuals with type 2 diabetes consistently exhibit higher plasma AVP concentrations, supporting a plausible mechanistic link between chronic LWI and metabolic dysregulation [9]. Importantly, sex-specific differences in AVP regulation have been reported, with females exhibiting lower osmotic thresholds for AVP release and greater hormonal modulation (e.g., by estrogen and progesterone) compared to males, which may contribute to variability in hydration and metabolic responses [10]. In addition, activation of the renin–angiotensin–aldosterone system in response to LWI or sustained water loss can impair insulin signaling via elevated aldosterone concentrations [11], while chronic alterations in cellular hydration status, especially cell shrinkage, due to osmotic and ion flux changes, can modulate intracellular ion composition, cytoskeletal organization, vesicular pH, and gene expression, thereby shifting cellular metabolism toward pathways associated with insulin resistance [12]. Furthermore, LWI may indirectly influence metabolic health by modulating dietary glycemic load, as water consumption with meals can attenuate postprandial glycemic responses [13].

Importantly, recent experimental evidence extends these observations to stress physiology. Kashi et al. [14] demonstrated that individuals with habitual LWI (~ 1.3 L·day^−1^) exhibit greater cortisol reactivity to acute psychosocial stress, as assessed by the Trier Social Stress Test, when compared to individuals with a regular high WI (~ 4.4 L·day^−1^). Despite similar increases in heart rate and subjective anxiety, only LWI individuals showed significant elevations in salivary cortisol following the stressor, indicating heightened hypothalamic–pituitary–adrenal (HPA) axis activation. Moreover, cortisol reactivity was positively associated with objective markers of hydration status, such as urine osmolality and urine color, with more concentrated urine reflecting greater stress-induced endocrine responses. These findings suggest that chronic underhydration may not only influence metabolic and renal pathways but also amplify neuroendocrine stress responses, providing a potential additional mechanism linking habitual LWI to adverse long-term health outcomes.

Despite these observations, interventions designed to increase 24-h WI remain scarce and are typically of short duration (most commonly lasting approximately 2–12 weeks), as demonstrated by a systematic review [15] of randomized clinical trials, which found that evidence linking changes in water intake to health-related outcomes is limited and heterogeneous, particularly in athletic populations. Notably, short-term interventions aimed at increasing water intake in athletes with low habitual intake have been conducted, although data remain unpublished [16]. This gap is notable given that a substantial proportion of athletes are habitually classified as low drinkers, consuming water volumes below recommended intake levels [7]. For instance, in a cross-sectional study [7] involving competitive athletes, habitual water intake was estimated using 7-day dietary records collected during their usual training and daily routines in the week preceding physiological assessments. Under these real-life conditions, 58% of participants were classified as low drinkers (consuming < 35 mL·kg^−1^·day^−1^). This study further compared athletes with different habitual water intakes by measuring total body water, intra- and extracellular water using gold-standard isotope dilution techniques. The data demonstrated that total body water and body fluid compartments can remain within normal ranges in LWI athletes. However, this apparent maintenance of body water masks a consistent finding: athletes with LWI exhibit more concentrated urine, indicating increased renal concentrating stress. Furthermore, athletes in the low water intake group were significantly younger than those in the high intake group (19.4 ± 5.2 vs. 22.4 ± 4.8 years, *p* = 0.033). While causality was not established, this observation may indicate that younger athletes are less experienced in managing hydration in the context of training, reinforcing the potential importance of targeted education as training loads and sweat losses increase. In fact, some literature has shown that educational interventions targeting younger athletes are effective in improving hydration status [17].

Although AVP concentration was not directly measured in the previous study [7], a more recent experimental study [18] in underhydrated athletes showed that, despite normal serum osmolality, these individuals exhibit elevated circulating AVP concentrations alongside increased urine concentration, consistent with patterns previously described in the non-athletic population [4]. Together, these findings strengthen the evidence that normal plasma osmolality may be maintained at the cost of increased endocrine (AVP-mediated) and renal stress in both athletic and non-athletic populations. Consistent with findings in athletes, evidence from non-athletic populations suggests that normal total body water and plasma osmolality values may not fully capture alterations in renal or endocrine function [4]. Thus, although athletes and clinicians have traditionally prioritized water intake from a performance perspective, there is a clear need to investigate the relationship between chronic LWI and long-term health in athletes, particularly given the consistently large water and electrolyte losses associated with training and competition.

Within this conceptual framework, evidence by Johnson et al. [11] from non-athletic populations indicates that a short-term increase in daily water intake, implemented over a period of four consecutive days, is sufficient to improve urinary hydration biomarkers and to significantly reduce circulating AVP concentration. However, whether comparable endocrine adaptations can be achieved in athletic populations following similarly short-term interventions (e.g., 4-day increases of water intake) remains uncertain. It is therefore plausible that, due to the greater physiological demands on fluid-regulatory mechanisms, the duration and/or magnitude of water intake interventions required to elicit a meaningful reduction in AVP secretion in athletes may need to exceed those effective in non-athletic individuals. This consideration highlights the need for appropriately designed intervention studies to clarify the hydration-endocrine responses and the potential health implications of chronic LWI in athletic populations.

Thus, it is evident that two key questions remain unanswered: “What are the long-term health risks for athletes who habitually consume low amounts of water?” and “Could these athletes benefit from increased 24-h water intake?” Although athletes are often perceived as protected from metabolic disease during their competitive years, this concept may be unwarranted. The absence of longitudinal studies examining habitual drinking patterns and long-term renal or metabolic health outcomes in athletes represents a critical gap in the literature. In the context of precision medicine, total daily water intake should be recognized not only as a performance variable but also as a modifiable lifestyle factor with potential long-term health implications for athletes. Future research should (a) explicitly address whether athletes with chronic LWI exhibit early markers of renal or metabolic dysfunction later in life, (b) determine the duration and magnitude of increased water intake required to normalize plasma AVP and urinary biomarkers in athletic populations, (c) evaluate whether hydration guidelines for athletes should incorporate health-based recommendations, and (d) assess the long-term sustainability of increased water intake following interventions, including whether behavioral changes are maintained after the intervention period. We propose that expanding the scope of such research beyond athletic performance represents a necessary evolution in sport nutrition, aligning athletic practice with contemporary public health and preventive medicine priorities.
